# Supernumerary Teeth in Primary Dentition and Early Intervention: A Series of Case Reports

**DOI:** 10.1155/2012/614652

**Published:** 2012-07-22

**Authors:** Rakesh N. Bahadure, Nilima Thosar, Eesha S. Jain, Vidhi Kharabe, Rahul Gaikwad

**Affiliations:** ^1^Department of Pedodontics and Preventive Dentistry, Sharad Pawar Dental College, Sawangi Meghe, Wardha 442005, India; ^2^Department of Pedodontics and Preventive Dentistry, F.O.D.S, C.S.M. Medical University, Lucknow 226003, India; ^3^Department of Public Health Dentistry, Sharad Pawar Dental College, Sawangi Meghe, Wardha 442005, India

## Abstract

Supernumerary teeth are considered as one of the most significant dental anomalies during the primary and early mixed dentition stages. They are of great concern to the dentists and parents because of the eruption, occlusal, and esthetic problems they can cause. Supernumerary teeth occur more frequently in the permanent dentition but rarely in primary dentition. Mesiodens is the most common type of supernumerary teeth but rarely seen in lower arch. Early recognition and diagnosis of supernumerary teeth is important to prevent further complications in permanent dentition. Four cases of supernumerary teeth with mesiodens in upper and lower arch in primary dentition and their management have been discussed.

## 1. Introduction

Supernumerary teeth are defined as any teeth or tooth substance in excess of the usual configuration of twenty deciduous and thirty-two permanent teeth. Classification of supernumerary teeth may be on the basis of position or form [1]. Supernumerary teeth may, therefore, vary from a simple odontoma, through a conical or tuberculate tooth, to a supplemental tooth which closely resembles a normal tooth. Supernumerary teeth are more frequently observed in permanent dentition than in deciduous dentition with predilection for the upper arch than lower arch in a proportion of 10 : 1 [2]. The prevalence of supernumerary teeth is 0.15–1% in permanent dentition [2] and 0.3–0.6% in the primary dentition [3] with predilection of 2 : 1 for male sex.

Supernumerary teeth may cause the delayed or impaired eruption of succedaneous teeth (26–52%), displacement or rotation of permanent teeth (28–63%), crowding, abnormal diastema, or premature space closure, dilaceration or abnormal root development of permanent teeth, cyst formation (4–9%), or eruption into nasal cavity [4]. Thus, early recognition and management is important as a preventive measure for permanent dentition.

## 2. Cases Presentation

### 2.1. Mesiodens in Primary Dentition

#### 2.1.1. Case**  **1

A 6-year-old male child had reported with the chief complaint of dental decay. The patient's medical history and family history were noncontributory.

**Figure 1:**
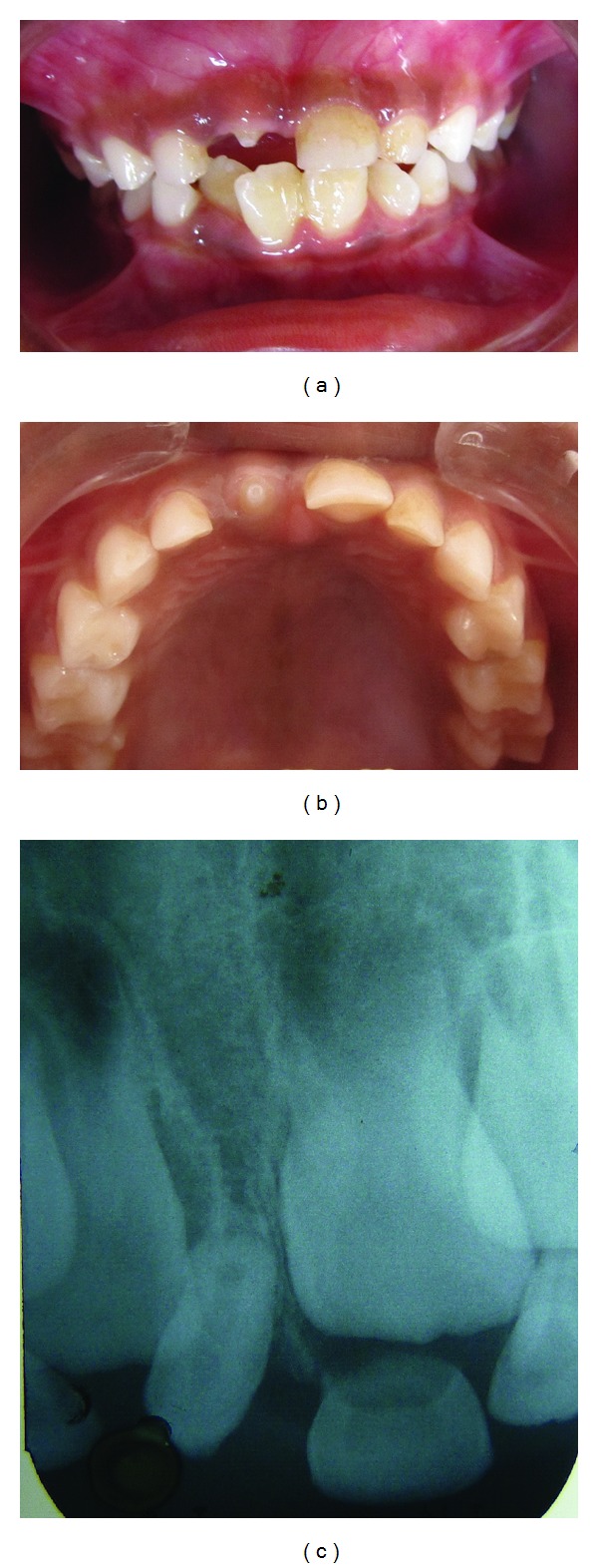
(a) Showing erupting mesiodens in place of central incisor, (b) showing occlusal view of mesiodens, and (c) radiograph showing mesiodens displacing permanent central incisor.

Intraoral examination showed missing right primary central incisor and presence of mesiodens erupting into the oral cavity in the same place (Figures 1(a) and 1(b)).

Intraoral periapical radiograph revealed deviation of 11 slightly due to presence of mesiodens and overlapping of 11 over 12 was observed (Figure 1(c)).

Also primary central incisor was exfoliated due to presence of mesiodens and mesiodens was not completely erupted. Only 2–2.5 mm of crown of mesiodens was visible intraorally. The mesiodens was responsible for deviation of permanent central incisor but was not extracted. Patient was kept under observation till the eruption of permanent right central incisor.

#### 2.1.2. Case**  **2

**Figure 2:**
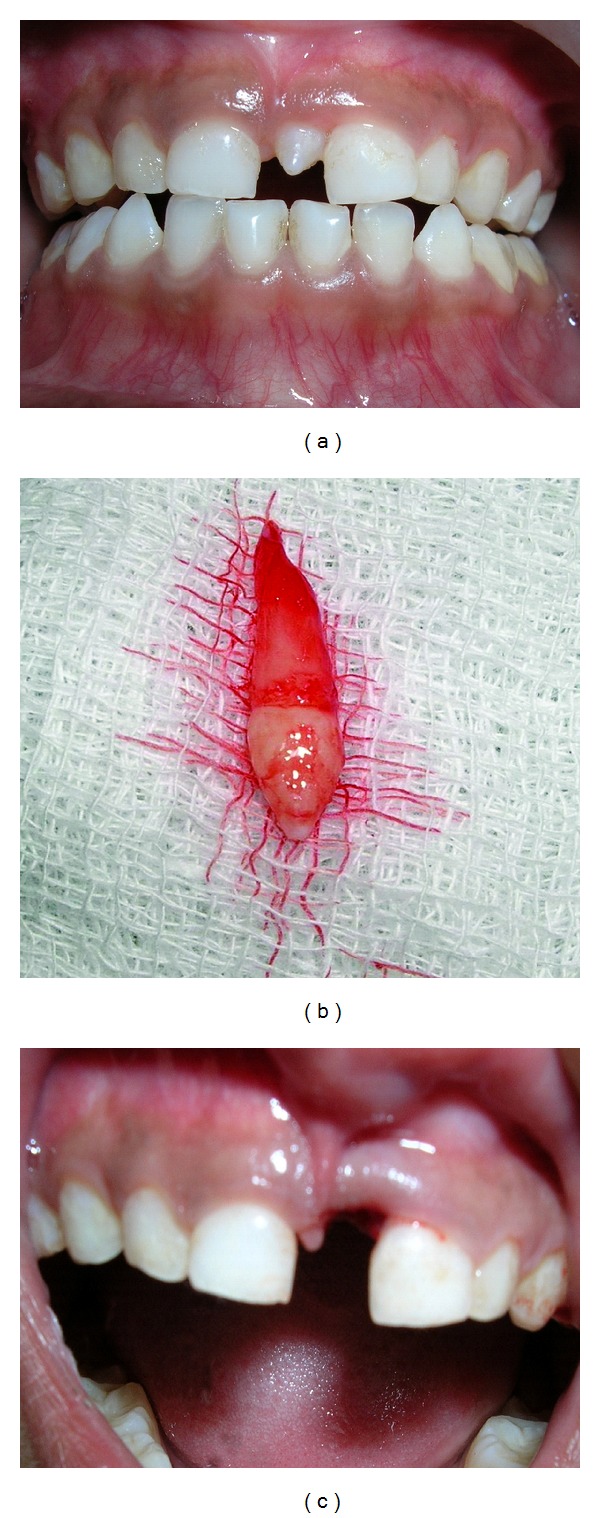
(a) Showing mesiodens erupting in between upper central incisors, (b) showing unilateral position of mesiodens socket, and (c) showing extracted mesiodens.

A 6-years-old male child was reported for dental check up. On intraoral examination, mesiodens was seen (Figure 2(a)).

Intraoral periapical radiograph of the patient showed that presence of mesiodens did not affect the position of permanent central incisors. Mesiodens was conical in shape with long root. Considering that the presence of mesiodens will be responsible for noneruption of permanent central incisors, as a precautionary measure, it was extracted. (Figures 2(b) and 2(c)).

Patient was kept under observation till the successful eruption of permanent central incisors.

#### 2.1.3. Case**  **3

**Figure 3:**
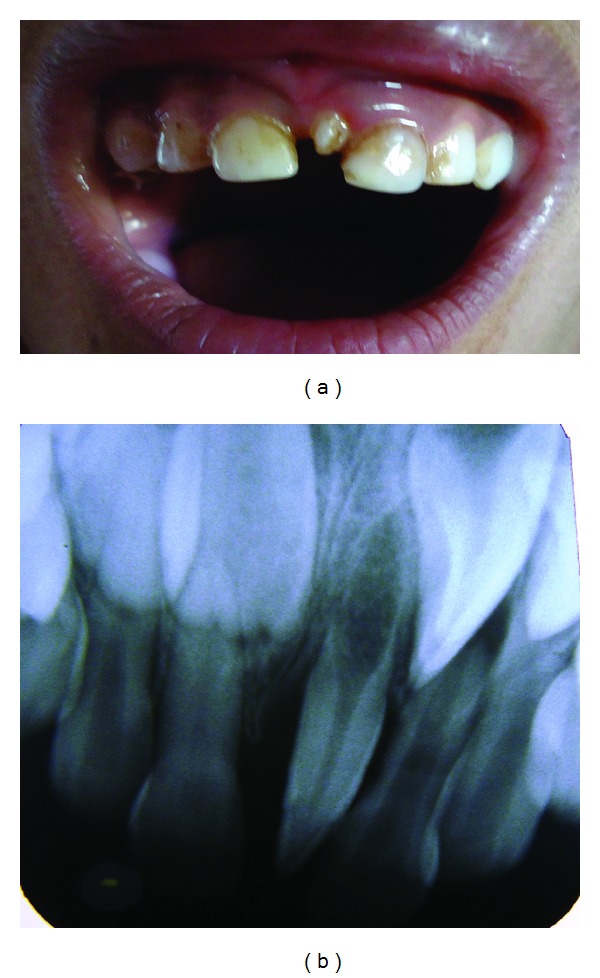
(a) Showing mesiodens in upper arch. (b) Radiograph showing rotated mesiodens and permanent incisor.

A 5-year-old male patient was reported for the dental checkup. On examination mesiodens was seen in upper arch (Figure 3(a)).

Patient was very uncooperative. Radiographically, it was rotated and only crown portion was formed. It was conical in shape (Figure 3(b)).

Considering that the presence of mesiodens may be responsible for noneruption of permanent central incisors, thus, as a precautionary measure, it was kept on regular followup so that preventive or interceptive measures can be applied at right time.

#### 2.1.4. Case**  **4

A 4-year-old female patient reported with the chief complaint of unerupted lower front teeth. Patient's medical history revealed that patient was a postoperated case of complete midline cleft palate, treated at the age of one year. Family history of the child was noncontributory.

**Figure 4:**
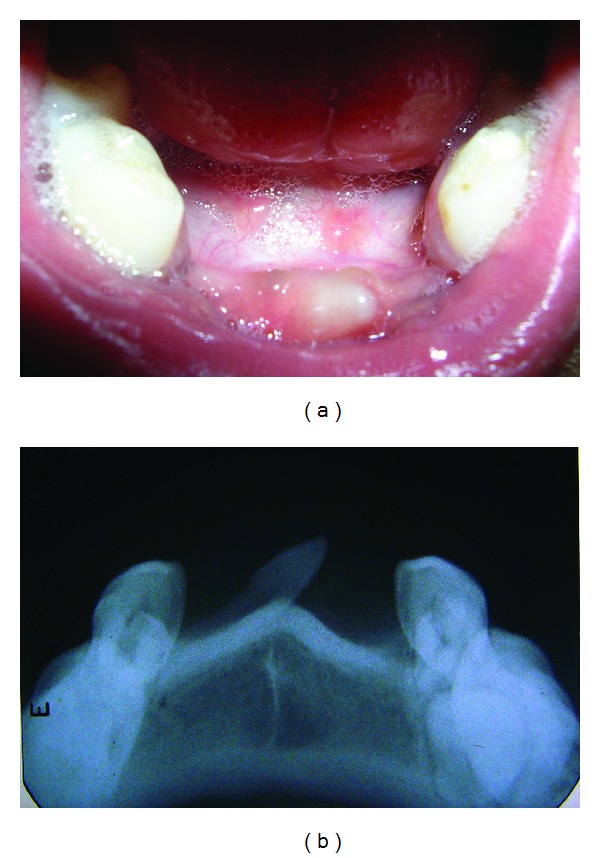
(a) Showing mesiodens in lower arch. (b) Radiograph showing mesiodens in lower arch.

Oral examination showed missing mandibular incisors and canines with an embedded supernumerary tooth in the midline (Figure 4(a)).

Anterior occlusal radiograph showed missing permanent mandibular tooth buds of 31, 32, 33, 41, 42, and 43 in lower anterior region with an embedded and obliquely placed supernumerary tooth which was parallel to the contour of the alveolar ridge with conical crown facing towards left side and long root facing towards right side of the mandibular arch (Figure 4(b)).

The treatment was aimed at addressing the patient's need for improved speech and aesthetics. So the embedded supernumerary tooth was extracted.

## 3. Discussion

Supernumerary teeth are less common in the deciduous dentition with a reported incidence of 0.3–0.6 percent of the population. Possible explanations for the less frequent reporting of deciduous supernumerary teeth include less detection by parents, as the spacing frequently encountered in the deciduous dentition may be utilized to allow the supernumerary tooth or teeth to erupt with reasonable alignment. Also, many children have an initial dental examination following eruption of the permanent anterior teeth. So anterior deciduous supernumerary teeth which have erupted and exfoliated normally would not be detected.

Supplemental teeth are less common than supernumerary teeth and are often overlooked because of their normal shape and size. Supplemental teeth may cause esthetic problems, delayed eruption, and crowding, and they require early diagnosis and treatment to prevent complications. Usually it is difficult to distinguish the normal tooth from its supplemental twin. Supplemental supernumerary teeth should be observed till the child is old enough, if it is not interfering with the development and eruption of adjacent teeth. Removal of supernumerary teeth is recommended in cases where they are causing any pathological changes or crowding along with esthetical problem and difficulty in oral hygiene maintenance. In the present case, since their presence did not cause esthetic problem nor was considered responsible for delayed eruption of permanent incisors as the case was reported earlier at 5 years of age, they were not extracted but maintained in the arch. Patients were kept under observation till the eruption of permanent incisors.

Multiple supernumerary teeth are a feature of certain disorders like cleidocranial dysplasia, cleft Palate, and Gardner's syndrome [5]. Detection of these teeth is best achieved by clinical and radiographic examination. An anterior occlusal radiograph is useful in locating a mesiodens. The management depends upon the type and position of such teeth and their effects on adjacent teeth. In one of our cases, in anterior occlusal radiograph, mesiodens was located in the midline of mandibular arch which was embedded in oblique direction. Intraorally presence of embedded mesiodens gave unaesthetic appearance to the patient and also would have interfered with placement of esthetic semifixed appliance, hence it was extracted.

## 4. Conclusion

The presence of supernumerary teeth is not uncommon in primary dentition and observed in both arches. But treatment varies to that of permanent dentition, depends upon the age of patient, cooperation on dental chair, and position of supernumerary tooth and their effects. It also depends on the length and size of supernumerary teeth and physiological resorption produced by erupting permanent tooth.

**Why This Paper Is Important to Pediatric Dentists.**

- Clinical and radiographic evaluation of supernumerary teeth should always be thorough in order to detect their presence.
- It is a great challenge to the clinicians to decide timely management of supernumerary teeth, to prevent complications associated with it.
- The reported data of supernumerary in primary dentition is less. We have to evaluate thoroughly the dental anomalies in primary dentition.
